# Supplementary material for: Antenatal intimate partner violence and breastfeeding practices: Evidence from a national longitudinal study in Ethiopia
Source: PLoS One. 2026 Apr 24;21(4):e0347323. doi: 10.1371/journal.pone.0347323 (PMC13108867; doi:10.1371/journal.pone.0347323)
Supplement: S1 Table — (DOCX) [file pone.0347323.s001.docx]

| Variable | Question Items and Responses  (0 = No, 1 = Yes) | Categorization criteria |
| --- | --- | --- |
| AN-IPV (physical and sexual) | Sometimes conflict can occur in relationships. At any time during pregnancy and/or the postpartum period, did your husband/partner do any of the following to you? A. Push you, shake you, or throw something at you?  B. Slap you? C. Twist your arm or pull your hair? D. Punch you with his fist or with something that could hurt you? E. Kick you, drag you, or beat you up? F. Try to choke you or burn you on purpose? G. Threaten or attack you with a knife, gun, or other weapon? H. Physically force you to have sexual intercourse with him when you did not want to? I. Physically force you to perform any other sexual acts you did not want to? J. Used threats or pressure to make you have sex when you didn’t want to, but did not use physical force? | Yes (experienced P-IPV): Responded "Yes" to at least one of the ten items during pregnancy. No (did not experience P- IPV): Responded "No" to all ten items during pregnancy and/or the postpartum period. |
