# Supplementary material for: Antenatal intimate partner violence and breastfeeding practices: Evidence from a national longitudinal study in Ethiopia
Source: PLoS One. 2026 Apr 24;21(4):e0347323. doi: 10.1371/journal.pone.0347323 (PMC13108867; doi:10.1371/journal.pone.0347323)
Supplement: S2 Table — (DOCX) [file pone.0347323.s002.docx]

| **Covariates** | | **EBF** | | **EIBF** | |
| --- | --- | --- | --- | --- | --- |
|  |  | **Yes (n, %)** | **No (n, %)** | **Yes (n, %)** | **No (n, %)** |
| **Partner encouragement for ANC and PNC** | None | 157 (74.92) | 53 (25.08) | 146 (69.65) | 64 (30.35) |
|  | One service  (ANC or PNC) | 219 (74.92) | 73 (25.08) | 211(72.21) | 81(27.79) |
|  | Both services (ANC and PNC) | 820 (75.97) | 259 (24.03) | 798 (73.93) | 282 (26.07) |
| Family size | 1-3 Members | 431 (73.94) | 151 (26.06) | 413 (70.91) | 170 (29.09) |
|  | 4-5 members | 434 (75.66) | 140 (24.34) | 431 (75.21) | 142 (24.79) |
|  | 6-13 members | 344 (75.67) | 110 (24.33) | 323 (71.03) | 132 (28.97) |
| Partner's educational status | No education | 346 (78.35) | 96 (21.65) | 301(68.02) | 141(31.98) |
|  | Primary | 499 (73.76) | 177 (26.24) | 504 (74.51) | 172 (25.49) |
|  | Secondary and higher | 363 (73.84) | 129 (26.16) | 363 (73.68) | 130 (26.32) |
| The partner has multiple wives | Yes | 98 (72.80) | 37 (27.20) | 95 (70.85) | 39 (29.15) |
|  | No | 1110 (75.32) | 364 (24.68) | 1072 (72.76) | 401 (27.24) |
| Feelings about the current pregnancy | Happy | 103 (57.21) | 77 (42.79) | 123 (68.58) | 57 (31.42) |
|  | Neutral | 163 (78.86) | 44 (21.14) | 160 (77.20) | 47 (22.80) |
|  | Unhappy | 934 (76.99) | 279 (23.01) | 876 (72.17) | 338 (27.83) |
| Number of marital unions | Married once | 1077 (74.74) | 364 (25.26) | 1055 (73.23) | 386 (26.77) |
|  | Married more than once | 131 (77.63) | 38 (22.37) | 112 (66.06) | 57 (33.94) |
| Postpartum contraceptive use at 6 weeks | Yes | 109 (66.42) | 55 (33.58) | 118 (72.01) | 46 (27.99) |
|  | No | 1099 (76.02) | 347 (23.98) | 1049 (72.53) | 397 (27.47) |
| Type of pregnancy (Singleton/Multiple) | Singleton | 1203 (75.97) | 379 (24.03) | 1152 (72.75) | 431 (27.25) |
|  | Twin | 6 (21.32) | 22 (78.68) | 16 (56.82) | 12 (43.18) |
| Number of ANC visits with HEW | No visit | 878 (73.15) | 322 (26.85) | 843 (70.24) | 357 (29.76) |
|  | 1-3 visits | 262 (79.64) | 67 (20.36) | 261 (79.41) | 68 (20.59) |
|  | 4+ visits | 69 (84.50) | 13 (15.50) | 63 (77.56) | 18 (22.44) |
| Number of ANC Visits with HCPs | No visit | 318 (74.00) | 112 (26.00) | 284 (66.17) | 145 (33.83) |
|  | 1-3 visits | 503 (78.00) | 142 (22.00) | 486 (75.41) | 158 (24.59) |
|  | 4+ visits | 388 (72.34) | 149 (27.66) | 397 (73.99) | 140 (26.01) |
| Experience of danger signs during pregnancy | Yes | 704 (75.01) | 234 (24.99) | 464 (69.04) | 208 (30.96) |
|  | No | 505 (75.09) | 167 (24.91) | 703 (74.94) | 235 (25.06) |
| Presence of obstetrics complication | Yes | 394 (73.54) | 141 (26.46) | 336 (62.64) | 200 (37.36) |
|  | No | 815 (75.79) | 260 (24.21) | 832 (77.38) | 243 (22.62) |
| Child sex | Male | 604 (74.57) | 206 (25.43) | 590 (72.78) | 221 (27.22) |
|  | Female | 604 (76.45) | 186 (23.55) | 577 (73.05) | 213 (26.95) |
| PNC attendance | Yes | 520 (74.72) | 176 (25.28) | 512 (73.57) | 184 (26.43) |
|  | No | 688 (75.29) | 226 (24.71) | 655 (71.64) | 259 (28.36) |
| PNC within 1 hour after birth | Yes | 195 (74.12) | 68 (25.88) | 192 (72.71) | 72 (27.29) |
|  | No | 1013 (75.22) | 334 (24.78) | 976 (72.43) | 371 (27.57) |
| Use of maternity waiting home | Yes | 117 (74.62) | 40 (25.38) | 106 (67.94) | 50 (32.06) |
|  | No | 1092 (75.09) | 361 (24.91) | 1061 (72.96) | 393 (27.04) |
| Crying at birth (newborn) | Yes | 1178 (76.51) | 361 (23.49) | 1143 (74.26) | 396 (25.74) |
|  | No | 31 (43.24) | 40 (56.76) | 24 (33.64) | 47 (66.36) |
| Belief: 'If I use family planning, my partner may seek another wife | Agree | 135 (74.88) | 45 (25.12) | 120 (66.87) | 60 (33.13) |
|  | Disagree | 1054 (74.99) | 352 (25.01) | 1038 (73.80) | 368 (26.20) |
| Skin-to-skin contact after birth | Yes | 650 (75.53) | 210 (24.47) | 684 (79.52) | 176 (20.48) |
|  | No | 559 (74.48) | 191 (25.52) | 483 (64.40) | 267 (35.60) |
| Partner’s age | 20-29 years | 398 (75.18) | 131 (24.82) | 403 (76.17) | 126 (23.83) |
|  | 30-39 years | 529 (74.91) | 177 (25.09) | 518 (73.28) | 189 (26.72) |
|  | 40-49 years | 199 (73.62) | 71 (26.38) | 178 (66.10) | 92 (33.90) |
|  | 50-97 years | 71 (76.50) | 22 (23.50) | 55 (59.98) | 37 (40.02) |
| LAM use intention | Yes | 286 (76.58) | 87 (23.42) | 297 (79.66) | 76 (20.34) |
|  | No | 736 (73.31) | 268 (26.69) | 695 (69.13) | 310 (30.87) |
| ANC counselling on PPFP | Yes | 132 (74.31) | 46 (25.69) | 136 (76.12) | 43 (23.88) |
|  | No | 879 (76.53) | 270 (23.47) | 854 (74.32) | 295 (25.68) |

ANC, Antenatal Care; PNC, Postnatal Care; PPFP, Postpartum Family Planning; LAM, Lactational Amenorrhea Method. Danger signs during pregnancy; presence of any of the following self-reported danger signs: severe or migraine headache, blurred vision, high blood pressure, edema involving the face and feet, convulsion/fits, and vaginal bleeding.

Obstetrics complications were defined as the presence of any of the following self-reported conditions: bleeding, membrane rupture before labor, membrane rupture at <9 months of gestation, malpresentation/malposition, prolonged labor (>12 hr), convulsion, retained placenta (>30 min), high fever with foul-smelling discharge or lower abdominal pain.
