# Supplementary material for: Antenatal intimate partner violence and breastfeeding practices: Evidence from a national longitudinal study in Ethiopia
Source: PLoS One. 2026 Apr 24;21(4):e0347323. doi: 10.1371/journal.pone.0347323 (PMC13108867; doi:10.1371/journal.pone.0347323)
Supplement: Attachment — Submitted filename: Response to Reviewers.docx [file pone.0347323.s005.docx]

**Authors’ response to editor and reviewers’ comments**

Dear Editor and reviewers,

Thank you for your constructive comments and insightful suggestions on our manuscript. Your expertise has been invaluable in improving our work. We have revised the manuscript based on your feedback. Please see below in blue for a point-by-point response to your comments and suggestions. All page numbers refer to the revised clean version of the manuscript file.

**Journal Requirements:**

When submitting your revision, we need you to address these additional requirements.

1. Please ensure that your manuscript meets PLOS ONE's style requirements, including those for file naming. The PLOS ONE style templates can be found at [https://journals.plos.org/plosone/s/file?id=wjVg/PLOSOne_formatting_sample_main_body.pdf](https://url.au.m.mimecastprotect.com/s/GYbeCANpDDtNrp5JksMuWHGYk2L?domain=journals.plos.org) and [https://journals.plos.org/plosone/s/file?id=ba62/PLOSOne_formatting_sample_title_authors_affiliations.pdf](https://url.au.m.mimecastprotect.com/s/Lo-2CBNqBBt7PWNJYUvCZH2g311?domain=journals.plos.org)

**Authors’ response:** We have ensured that our manuscript now complies with all formatting and file naming requirements.

2. Please amend your authorship list in your manuscript file to include author [Solomon Abrha](mailto:abrsole2006@gmail.com).

**Authors’ response:** The authorship list has been updated to include Solomon Abrha Damtew.

3. Please amend the manuscript submission data (via Edit Submission) to include author Solomon Abrha Damtew.

**Authors’ response**: We have amended the manuscript submission data, and the author Solomon Abrha Damtew has been included.

4 Please include a separate caption for each figure in your manuscript.

**Authors’ response**: We have included a separate caption for each figure in the revised manuscript as requested (See figure legends below the reference, page 26, lines 724-732).

5. We note that Figure(s) 2, 3, 4 in your submission contain copyrighted images. All PLOS content is published under the Creative Commons Attribution License (CC BY 4.0), which means that the manuscript, images, and Supporting Information files will be freely available online, and any third party is permitted to access, download, copy, distribute, and use these materials in any way, even commercially, with proper attribution. For more information, see our copyright guidelines: [http://journals.plos.org/plosone/s/licenses-and-copyright](https://url.au.m.mimecastprotect.com/s/HFmnCD1vkkT5jYZr4fBFAHj75m8?domain=journals.plos.org).
We require you to either (1) present written permission from the copyright holder to publish these figures specifically under the CC BY 4.0 license, or (2) remove the figures from your submission:
a. You may seek permission from the original copyright holder of Figure(s) 2, 3, 4 to publish the content specifically under the CC BY 4.0 license.
We recommend that you contact the original copyright holder with the Content Permission Form ([http://journals.plos.org/plosone/s/file?id=7c09/content-permission-form.pdf](https://url.au.m.mimecastprotect.com/s/5GJyCE8wRRt314joECyHLH7WPrw?domain=journals.plos.org)) and the following text:
“I request permission for the open-access journal PLOS ONE to publish XXX under the Creative Commons Attribution License (CCAL) CC BY 4.0 ([http://creativecommons.org/licenses/by/4.0/](https://url.au.m.mimecastprotect.com/s/l6rhCGv066U1W39ZvfWIgHBFBKT?domain=creativecommons.org)). Please be aware that this license allows unrestricted use and distribution, even commercially, by third parties. Please reply and provide explicit written permission to publish XXX under a CC BY license and complete the attached form.”
Please upload the completed Content Permission Form or other proof of granted permissions as an "Other" file with your submission.
In the figure caption of the copyrighted figure, please include the following text: “Reprinted from [ref] under a CC BY license, with permission from [name of publisher], original copyright [original copyright year].”
b. If you are unable to obtain permission from the original copyright holder to publish these figures under the CC BY 4.0 license or if the copyright holder’s requirements are incompatible with the CC BY 4.0 license, please either i) remove the figure or ii) supply a replacement figure that complies with the CC BY 4.0 license. Please check copyright information on all replacement figures and update the figure caption with source information. If applicable, please specify in the figure caption text when a figure is similar but not identical to the original image and is therefore for illustrative purposes only.

**Authors’ response**: We would like to confirm that all figures included in the manuscript were **generated directly from our original data. Figure 2** was created using **DAGitty software**, while **figures 3 and 4** were generated using **Stata**. These are entirely original figures created by the authors, with no content copied from third-party sources. Therefore, no third-party copyright permission is required, and the figures can be published under the CC BY 4.0 license.

6. [Please include captions for your Supporting Information files at the end of your manuscript, and update any in-text citations to match accordingly. Please see our Supporting Information guidelines for more information: http://journals.plos.org/plosone/s/supporting-information.](https://url.au.m.mimecastprotect.com/s/eoPsCJyB66iqp9oLNUnS1HyflSK?domain=track.editorialmanager.com)

**Authors’ response**: We have included captions for all supporting information files at the end of the manuscript (See supporting information below the reference, page 26, lines 733-737).

7. We note that there is identifying data in the Supporting Information file <Original Ethical approval letter.pdf>. Due to the inclusion of these potentially identifying data, we have removed this file from your file inventory. Prior to sharing human research participant data, authors should consult with an ethics committee to ensure data are shared in accordance with participant consent and all applicable local laws.
Data sharing should never compromise participant privacy. It is therefore not appropriate to publicly share personally identifiable data on human research participants. The following are examples of data that should not be shared:
-Name, initials, physical address
-Ages more specific than whole numbers
-Internet protocol (IP) address
-Specific dates (birth dates, death dates, examination dates, etc.)
-Contact information such as phone number or email address
-Location data
-ID numbers that seem specific (long numbers, include initials, titled “Hospital ID”) rather than random (small numbers in numerical order)

Data that are not directly identifying may also be inappropriate to share, as in combination they can become identifying. For example, data collected from a small group of participants, vulnerable populations, or private groups should not be shared if they involve indirect identifiers (such as sex, ethnicity, location, etc.) that may risk the identification of study participants.
Additional guidance on preparing raw data for publication can be found in our Data Policy ([https://journals.plos.org/plosone/s/data-availability#loc-human-research-participant-data-and-other-sensitive-data](https://url.au.m.mimecastprotect.com/s/pEM_CK1DrrT287kxVfrTZH5_EAx?domain=journals.plos.org)) and in the following article: [http://www.bmj.com/content/340/bmj.c181.long](https://url.au.m.mimecastprotect.com/s/_1oPCL7E99TRNB2M6TkUYHyfxxh?domain=bmj.com).
Please remove or anonymize all personal information (<specific identifying information in file to be removed>), ensure that the data shared are in accordance with participant consent, and re-upload a fully anonymized data set. Please note that spreadsheet columns with personal information must be removed and not hidden as all hidden columns will appear in the published file.

**Authors’ response**: An anonymised dataset has been uploaded as requested.

8. If the reviewer comments include a recommendation to cite specific previously published works, please review and evaluate these publications to determine whether they are relevant and should be cited. There is no requirement to cite these works unless the editor has indicated otherwise. 
**Authors’ response**: Thanks for this. In our case, the reviewers did not recommend citing any specific previously published works. Therefore, no additional references were added in response to reviewer recommendations.
**Additional Editor Comments:**

**1. Data used were collected at baseline and 6 weeks after birth. Baseline can be during pregnancy to 6 weeks after birth, therefore duration from baseline to 6weeks time point can vary from zero days to more than 6 weeks (Fig 1). Is the exposure data (AN-IPV) collected at baseline and outcome data (EIBF and EBF) collected 6 weeks? How is the variability in this duration is accounted in the analysis?**

**Authors’ response:** We thank the editor for raising this important point. Both the exposure (AN-IPV) and the outcomes (EIBF and EBF) were assessed at the six-week follow-up interview. Although AN-IPV was measured at six weeks postpartum, women were specifically asked about their experiences of IPV **during pregnancy** using the question: "At any time during your pregnancy, did your husband/partner do any of the following things to you?" This was designed to capture IPV exposure throughout the entire pregnancy. Because both exposure and outcome variables were collected at the same six-week follow-up, variability in the timing of the baseline interview does not affect the temporal alignment between AN-IPV and the breastfeeding outcomes. However, this timing may introduce recall bias, which we have acknowledged in the Limitations section, as follows:

**See limitations section, page 17, lines 396–401, paragraph 1**.

*“Second,* *AN-IPV was assessed retrospectively at the six-week postpartum interview. Although the questions were specifically framed to refer to experiences during pregnancy, self-reported data may be subject to recall bias. Misclassification of AN-IPV exposure due to imperfect recall is possible; it is likely non-differential and may have affected the observed associations with breastfeeding outcomes (1).”*

**2. 1841 baby-mother pairs included in the analyses. That implies no twin births. But I noted having 28 twin births in results. Can EBF and/or EIBF differ between twins, if so how they were accounted in results?**

**Authors’ response:** We thank the reviewer for this thoughtful comment. We agree that EBF and EIBF may differ between twins. However, because the number of twin births was relatively small, we did not conduct a separate twin-specific analysis. In our study, breastfeeding outcomes were assessed at the **infant level;** therefore, each twin was treated as a separate observation. As the unit of analysis was the infant, the reported final sample size of **1,841** reflects the total number of infants included in the analyses, including those from 28 twin deliveries. To account for the non-independence of twins born to the same mother, we adjusted for clustering at the maternal level in our analyses. We also acknowledge that the phrase “baby–mother pairs” may have been misleading, and we have revised the wording to avoid ambiguity.

**See results section, page 10, line 298, paragraph 1.**

“*A total of 1,841 infants and their mothers were included in the study”…..*

**3. Box with 1799 women in Fig1 is confusing as it says 2072 women were resulted from 1799 women. Consider removing that box (2024+273-177-35-13=2072).**

**Authors’ response:** The box showing “1799 women” in Figure 1 has now been removed.

**Spellings:
4. table1, fig3, fig4 titles. Sex weeks to be changed to six weeks?
Fig4 axis title, should P-IPV to be changed to AN-IPV?.**

**Authors’ response:** These have been corrected.

**Review Comments to the Author**

**Reviewer #1**:

Great work overall, and it is good to see that a directed acyclic graph was used in this study. Please consider the following points regarding the results:

**1. There may be a need to reorganize Table 1. Since the table spans multiple pages, breaking it down into groups would enhance comprehension and prevent the meaning from getting lost.**

**Authors’ response:** We thank the reviewer for this helpful suggestion. Table 1 has been revised to improve clarity and readability. Less essential variables were moved to the supplementary material (See Table 1, results section, pages 11-12).

**2. It is understandable that the models were assessed for goodness of fit, but did the authors evaluate whether the variables included were appropriate for the model? This could affect the findings, particularly if the models contain too few, too many, or irrelevant variables. It would be helpful to have the full model tables to see which variables were included.**

**Authors’ response:** We carefully evaluated the variables included in the models to ensure their appropriateness. The selection of variables was guided by the **directed acyclic graph (DAG)**, relevant literature, and our empirical knowledge of the study context (IPV and breastfeeding outcomes). To provide clarity, we have indicated the variables included in each model in the **footnotes of the corresponding tables (Tables 2 and 3).** We chose this approach because the main aim of the study is to examine the effects of AN-IPV on EIBF and EBF, and including all variables in the table would make the tables excessively lengthy (as seen in Table 1) and potentially reduce readability.

**3. If the authors included “any form of violence” and then also included physical and sexual violence separately in the same model, could that affect the outcome? It is unclear whether the authors considered this. “Any AN-IPV” was significant in the final model, but the separate violence categories (physical and sexual) were not; why? Would the authors consider a model with interaction terms? What other factors could be influencing these models? Should the authors have included only physical and sexual IPV rather than “any AN-IPV”? This distinction is important for public health actions and recommendations, as stating that “any AN-IPV affects EIBF” may be too vague.
Additionally, more rigorous analyses may be warranted**

**Authors’ response:** We thank the reviewer for these insightful comments regarding the modelling of AN-IPV. We provide the following clarifications:

1. Modelling of “any AN-IPV” vs. separate forms (physical and sexual AN-IPV):
   To evaluate the effects of AN-IPV comprehensively, we fitted separate multivariable multilevel logistic regression models for each exposure:
   - Any AN-IPV on EIBF and EBF
   - Physical AN-IPV on EIBF and EBF
   - Sexual AN-IPV on EIBF and EBF

For each model, the other forms of violence were not included as covariates. For example, in the model assessing the effect of “any AN-IPV,” physical and sexual AN-IPV were not included (**See methods section, page 9, lines 275-278**). This approach avoids collinearity and ensures that each model estimates the independent effect of the exposure of interest.

2. Interaction terms:
    Each exposure (physical and sexual, and any type of AN-IPV) was analysed in separate models; therefore, interaction terms between different forms of AN-IPV were not included.
3. Differences in significance:
   In the final models, “any AN-IPV” was significant while the separate forms (physical or sexual) were not in some cases. As the variable “any AN-IPV” captures all forms of violence collectively, it results in a higher exposure prevalence and therefore greater statistical power compared with specific forms (e.g., physical or sexual IPV), which may explain the variability in statistical significance.
4. Other covariates influencing the models:
   All relevant potential confounders were included according to our directed acyclic graph (DAG) and empirical knowledge. These variables are listed in the footnotes of the tables (Table 2), including maternal age, education, marital status, wealth index, partner characteristics, parity, pregnancy intention, place and mode of delivery, ANC visits, pregnancy complications, newborn sex, residence, and region.
5. Rationale for including “any AN-IPV”:
   We included “any AN-IPV” as the main exposure because our study aims to evaluate the overall impact of antenatal IPV on breastfeeding outcomes, which is critical for public health interventions. Separate analyses for physical and sexual AN-IPV were also conducted and reported, allowing readers to assess both the overall and specific effects.
6. Rigorous analyses:
   Multilevel logistic regression with adjusted covariates and clustering ensures robustness.

We hope these clarifications address the reviewer’s concerns.

**Reviewer #2:**

**Here is a list of specific comments. Note: page numbering in reviews and comments is based on ruler applied in Editorial Manager-generated PDF. There is no line numbering.**

**1. Page 3, line 6, “impact”: Because the study is an observational study, I recommend avoiding words with causal interpretation such as “impact,” “effects,” etc. throughout the manuscript.**
**Authors’ response:** Thank you for your constructive comments and insightful suggestions on our manuscript**.** We have revised this throughout the manuscript.

**2. Page 3, line 14, “accounting for the hierarchical structure of the data”: I recommend being more specific about what hierarchical structure is accounted in the multilevel models.**
**Authors’ response:** To clarify, the hierarchical structure accounted for in our multilevel models refers to **mothers (level 1) nested within clusters/communities (enumeration areas, level 2)**.

Accordingly, the revised sentence now reads:

**See methods section, page 9, lines 274-275.**

" *A two-level multilevel binary logistic regression model was employed, with mothers at level 1 nested within clusters (enumeration areas) at level 2*".

**3. Page 5, lines 2–3, “while some studies . . . ”: I recommend describing the direction, a negative or positive association, among references #22–28, as comparing to that in reference #21.**

**Authors’ response:** We have revised the text to clarify the direction of the associations as follows

**See the introduction section, Pages 4-5, lines 129-133, paragraph 4.**

**“**Furthermore, AN-IPV is believed to negatively affect maternal breastfeeding practices, including early initiation of breastfeeding (EIBF) and exclusive breastfeeding (EBF) (2-7). However, some studies found no significant association between AN-IPV and breastfeeding indicators (EIBF and EBF) (5, 8, 9), highlighting inconsistencies in the current body of literature.”

**4. Page 7, lines 5–6, “a binary variable, . . . ”: Because EBF is defined as a proportion, I recommend providing additional information regarding how the binary EBF is defined (e.g., the proportion >0 vs 0).**

**Authors’ response:** We have revised the methods section to clarify how the binary EBF variable was defined.

**See methods section, page 7, lines 216-218.**

“*For analysis, a binary variable was created: infants who received only breast milk in the past 24 hours were coded as ‘1’ (EBF), and infants who received any other foods or liquids were coded as ‘0’ (non-EBF*)”.

**5. Page 7, lines 16–18, “covariates included . . . ”: I recommend reminding readers that the manuscript** **assumes that both outcomes, EIBF and EBF, share the same DAG; i.e.,** **the same minimally sufficient set of variables are used in both EIBF and EBF models.**

**Authors’ response:** We have revised the Methods section to explicitly state that the manuscript assumes both outcomes (EIBF and EBF) share the same DAG, and that the same minimally sufficient adjustment set was used in both models.

**See methods section, page 8, lines 233-235.**

*“We assumed that the two outcomes, EIBF and EBF, share the same DAG; i.e., the same minimally sufficient set of variables was used in both EIBF and EBF models.”*

**6. Page 7, line 20, “these variables were grouped into five broad categories”: I recommend stating how many variables are included in the minimally sufficient set and adjusted in the models.**
**Authors’ response:** We have revised the Methods section to specify the number of variables included in the minimally sufficient adjustment set used in the multivariable models. In addition, the variables included in the multivariable models are listed as a footnote below the relevant table.

**See methods section, page 8, lines 251-252.**

*“In total, 15 variables comprised the minimally sufficient adjustment set and were included in the multivariable models.”*

**7. Page 8, line 3, “stratifier variable”: I recommend describing the enumeration areas as cluster variable and reserving stratifying variables for the AN-IPV types and urban/rural areas.**

**Authors’ response:** We have revised the manuscript to describe enumeration areas as the clustering variable in the multilevel models and clarified that stratification by AN-IPV type and urban/rural area/region was applied in the descriptive analyses.

**See methods section, page 8, lines 253-255.**

*“**Cluster variable: Enumeration areas (EAs) were included as the cluster variable in the multilevel models, with mothers nested within clusters (ranging from 1 to 162 EAs).*

*Stratified variables included AN-IPV types, urban/rural residence, and region.”*

**8. Page 8, lines 3–4, “cluster number . . . ”: I recommend revising “cluster number” as ‘****cluster identification.’**

**Authors’ response:** This has been corrected.

**See methods section, page 8, lines 253-254.**

*“Cluster variable: Enumeration areas (EAs) were included as the cluster variable in the multilevel models, with mothers nested within clusters (ranging from 1 to 162 EAs). “*

**9. Page 8, line 15, “homogeneity of variance”: Logistic regression does not require the homogeneity of variance assumption. I recommend removing it.**

**Authors’ response:** This has been corrected.

**10. Page 8, lines 23–24, “model fit . . . ”: The variables included in the EIBF and EBF models are determined by the DAG. Therefore, there is no variable selection process in the analysis. I recommend clarifying the selection of the best-fitting model using DIC.**

**Authors’ response:** We agree that because covariates in the EIBF and EBF models were determined a priori using a DAG, no data-driven variable selection was performed. We have revised the manuscript to clarify that the Deviance Information Criterion (DIC) was used solely to assess relative model fit among the sequentially specified models, rather than for variable selection.

**See methods section, page 9, lines 270-273.**

*“Model fit was assessed using the Deviance Information Criterion (DIC) to compare the sequential models. Because covariates were identified a priori using the DAG, DIC was used solely to evaluate relative model fit, not for variable selection*.”

**11. Page 8, line 26, “communities”: I recommend referring the clusters as the enumeration areas for consistency.**

**Authors’ response:** We have revised the terminology accordingly and now consistently refer to “communities” as “clusters (enumeration areas)” throughout the manuscript.

**See methods section, page 9, lines 274-275.**

*“A two-level multilevel binary logistic regression model was employed, with mothers at level 1 nested within clusters (enumeration areas) at level 2.”*

**12. Page 8, lines 26–32, “three models . . . ”: I see the rationale of DIC now. However, because the confounders have been identified via the DAG, associations in Model 1 and Model 2 are assumed biased, provided that the DAG is accurate. I will emphasize again the clarification of model selection and sequential models.**

**Authors’ response**: We agree that, under a DAG-informed framework, only the fully adjusted model is expected to provide minimally biased estimates, assuming the DAG is correctly specified. Accordingly, we have revised the manuscript to clarify that the models were fitted sequentially for transparency and to illustrate the impact of progressive adjustment. We now explicitly state that Model 3, which includes the full minimally sufficient adjustment set, was considered the primary inferential model. Models 1 and 2 are presented to illustrate the crude and partially adjusted associations for comparison.

**See methods section, page 9, lines 283-287.**

“*The models were specified sequentially to illustrate the influence of progressive adjustment. Model 3, which included the full minimally sufficient adjustment set, was considered the primary inferential model. Models 1 and 2 are presented to illustrate the crude and partially adjusted associations for comparison.”*

**13. Page 9, lines 12–13, “after applying sampling weights . . . ”: Please clarify in the manuscript the reason the weighted sample size, 1610, is smaller than 1841.**

**Authors’ response:** The weighted sample size (1610) is smaller than the unweighted sample (1841) because the sampling weights were applied to account for the survey design and non-response. The weighted sample reflects the **effective sample size** used in the analyses. We have clarified this in the revised manuscript.

**See results section, page 10, lines 298-300.**

“*After applying sampling weights to account for the survey design and non-response, the weighted sample size was 1,610, which reflects the effective sample size used in the analyses.”*

**Reviewer #3**:

**The authors used nationally representative data in Ethiopia to examine the association between intimate partner violence (IPV) and breastfeeding (BF). The results are reasonable, with two comments.**

**1. Why did the authors use iweight for descriptive statistics and pweight for regression models?**

**Authors’ response:** We thank the reviewer for helpful comments and suggestions. We have now applied pweight for both descriptive and regression analyses for consistency.

**See methods section, pages 8-9, lines 259-260.**

*“Sampling weights were applied using the* ***“****pweight****”*** *command for both descriptive and regression analyses*.”

**2. The authors acknowledged there might be under-reporting of IPV and over-reporting of BF. Instead of assuming the two reporting biases were independent, quantitative bias analyses are needed.**

**Authors’ response:** We acknowledge that under-reporting of IPV and over-reporting of breastfeeding, due to reliance on participants’ self-reports, could introduce misclassification bias. This misclassification is likely **non-differential and may affect** the observed associations. While a formal quantitative bias analysis was beyond the scope of the current study, we have acknowledged this limitation in the revised manuscript and discussed its potential impact on the results.

**See limitation section, page 17, lines 391-396, paragraph 1.**

*“First, both AN-IPV exposure and breastfeeding outcomes (EIBF and EBF) were based on mothers’ self-reports collected via interviewer-administered questionnaires, which may have introduced social desirability bias. This could have resulted in underreporting of AN-IPV experiences or overreporting of adherence to recommended breastfeeding practices,* ***potentially introducing misclassification bias****. Such misclassification is likely non-differential and may have affected the observed associations.”*

**Reviewer #4:**

This manuscript examines the association between antenatal intimate partner violence (AN-IPV) and breastfeeding practices using nationally representative longitudinal data from the PMA-Ethiopia survey. The topic is highly relevant to maternal and child health in low-resource settings, and the authors address an important evidence gap in Ethiopia. The use of a longitudinal cohort, multilevel modelling, and theory-driven confounder selection strengthens the methodological rigour of the study.

The manuscript is well written, methodologically sound, and suitable for publication in PLOS ONE. However, several issues related to conceptual clarity, measurement, interpretation of findings, and presentation should be addressed to improve the scientific clarity and robustness of the paper.
**Authors’ response**: We thank the reviewer for the constructive suggestions that will help improve the paper. We have carefully revised the manuscript and addressed each of the reviewer’s comments in detail below.
Major Comments
**1. Conceptual Framework and Mechanisms
The conceptual mechanisms linking IPV during pregnancy to breastfeeding outcomes are not sufficiently articulated. The introduction and discussion would benefit from a clearer theoretical explanation of how AN-IPV may differentially affect EIBF versus exclusive breastfeeding (EBF).**

**Recommendation: Include a brief conceptual discussion outlining plausible biological (e.g., stress-related hormonal disruption), psychological (e.g., trauma, depression), and social (e.g., partner control, reduced support) pathways linking AN-IPV to breastfeeding practices.**

**Authors’ response**: We have briefly highlighted in the introduction that AN-IPV may influence breastfeeding practices, including EIBF and EBF, through **biological** (stress-related hormonal disruption), **psychological** (depression, anxiety, trauma), and **social** (partner control, reduced support) pathways.

**See the introduction section, page 5, lines 134-148, paragraph 5.**

*“AN-IPV may influence breastfeeding practices such as EIBF and EBF through biological, psychological, and social pathways. Biologically, AN-IPV acts as a chronic stressor, triggering the hypothalamic-pituitary-adrenal (HPA) axis and elevating stress hormones such as cortisol and catecholamines. This dysregulation can interfere with hormonal processes vital for lactation, notably by altering prolactin and oxytocin levels—prolactin being essential for milk production and oxytocin for the milk ejection reflex. Such disruptions can impair milk synthesis and let-down, potentially delaying initiation and shortening EBF duration (10-12).*

*Psychologically, AN-IPV may cause adverse mental health effects such as depression, anxiety, and trauma-related distress, which can diminish maternal motivation, emotional availability, and breastfeeding self-efficacy—key factors for initiation and continuation. Women facing AN-IPV are at higher risk for mental disorders, and these conditions are linked to reduced confidence, feeding anxiety, and earlier EBF cessation (13-17). Trauma symptoms such as fear and hypervigilance may further hinder maternal–infant bonding and responsive feeding (18). Socially, IPV can restrict maternal autonomy and access to support networks due to controlling behaviours, reduced social and family support, and isolation. These factors limit practical support and access to healthcare, weakening the environmental conditions necessary for both EIBF and sustained EBF (19-22).”*

**2. Timing of Exposure and Outcome Measurement
Both exposure (AN-IPV) and outcomes (EIBF and EBF) were collected at the six-week postpartum interview. Although AN-IPV is defined as occurring during pregnancy, retrospective reporting at six weeks postpartum raises concerns about recall bias and temporal ambiguity.**

**Recommendation: Clarify how AN-IPV questions were framed to ensure they referred strictly to the pregnancy period. Discuss potential recall bias and its likely impact on effect estimates more explicitly in the limitations section.**

**Authors’ response:** We thank the reviewer and editor for this insightful and important comment regarding recall bias and temporal ambiguity. In response, we have clarified the AN-IPV measurement framing in the **methods section**. We also agree that retrospective reporting at six weeks postpartum may introduce **recall bias**. Accordingly, we have strengthened the **limitations section** to acknowledge this issue.

**See methods section, page 7, lines 224-226.**

“Postpartum women were specifically asked about their experiences of IPV during pregnancy using the question: ‘At any time during your pregnancy, did your husband/partner do any of the following things to you?’” This wording explicitly anchors the exposure to the pregnancy period, thereby reducing concerns about temporal ambiguity between exposure and outcomes.

**See limitations section, page 17, lines 396–401, paragraph 1.**

“*Second, AN-IPV was assessed retrospectively at the six-week postpartum interview. Although the questions were specifically framed to refer to experiences during pregnancy, self-reported data may be subject to recall bias. Misclassification of AN-IPV exposure due to imperfect recall is possible; it is likely non-differential and may have affected the observed associations with breastfeeding outcomes*.”

**3. Interpretation of Null Findings for Exclusive Breastfeeding

The manuscript reports no statistically significant association between AN-IPV and EBF at six weeks postpartum, yet this finding is not sufficiently explored. Given that EBF is a key outcome, the lack of association warrants deeper interpretation. Recommendation: Expand the discussion to explore possible explanations for the null association, such as the short follow-up period, strong breastfeeding norms in Ethiopia, measurement using 24-hour recall, or insufficient statistical power for IPV sub-types.**

**Authors’ response:** We appreciate the reviewer’s insightful comment. In the revised manuscript, we have expanded the discussion to explore possible explanations for the null association

**See discussion section, page 16-17, lines 375-387, paragraph 3.**

“Although our results indicated that AN-IPV was not significantly associated with EBF, this finding should be interpreted with caution. Several factors may explain the observed null association. First, the short follow-up period: EBF was assessed at six weeks postpartum rather than the WHO-recommended six months. Breastfeeding practices may change over time as maternal and infant factors evolve, and associations with AN-IPV may become more evident later in the postpartum period when sustained breastfeeding challenges arise (20).

Second, breastfeeding is nearly universal in Ethiopia (23). In contexts characterised by near-universal breastfeeding, social networks, perceived norms, and strong family and community expectations play a central role in shaping mothers’ breastfeeding practices and success, and may buffer potential adverse influences, including IPV (24). Third, EBF was measured using a 24-hour recall method, which may overestimate the true EBF practices (25, 26). Finally, the relatively small number of women reporting physical and sexual AN-IPV may limit statistical power, which could mask real associations.”

**4. Measurement of Intimate Partner Violence

The study excludes psychological/emotional IPV due to data limitations, which likely underestimates the true burden of AN-IPV and may bias associations toward the null.
Recommendation: Strengthen the limitations section by explicitly discussing how exclusion of emotional IPV may affect prevalence estimates and observed associations.**

**Authors’ response:** We have strengthened the limitations section to clarify that psychological/emotional AN-IPV was not measured, which likely underestimates prevalence. We now explicitly note that excluding emotional IPV may have introduced exposure misclassification, potentially biasing associations toward the null and attenuating true effects.

**See limitations section, page 17, lines 411-415.**

“*Excluding psychological/emotional AN-IPV may also have influenced the observed associations, as women exposed solely to psychological/emotional violence would have been misclassified as unexposed. This non-differential misclassification could bias estimates toward the null, potentially attenuating true associations between AN-IPV and breastfeeding outcomes.”*
**5. Statistical Power and Subgroup Analyses
The prevalence of sexual and physical AN-IPV is relatively low, which may limit statistical power, particularly in stratified analyses.**

**Recommendation: Acknowledge the potential for limited power to detect associations, especially in fully adjusted models for IPV sub-types.**

**Authors’ response:** We thank the reviewer for this important observation. We have now acknowledged in the discussion that the relatively low prevalence of sexual and physical AN-IPV may have limited statistical power, particularly in stratified and fully adjusted analyses of AN-IPV subtypes.

**See limitations section, page 18, lines 420-423.**

*“Sixth, the relatively low prevalence of sexual and physical AN-IPV in our sample may have limited statistical power to detect associations, particularly in stratified and fully adjusted models examining AN-IPV subtypes.”*

**Minor Comments**
**Length and Structure of Table 1
Table 1 is very detailed and may overwhelm readers. Suggestion: Consider moving some variables to supplementary material or highlighting key predictors in the text.**

**Authors’ response:** We thank the reviewers (reviewer 4 and reviewer 1) for this helpful suggestion. Table 1 has been revised to improve clarity and readability. Less essential variables were moved to the supplementary material.
**Abstract Conclusion
The conclusion of the abstract slightly overgeneralizes the findings.**

**Suggestion: Rephrase to clearly distinguish findings for EIBF versus EBF.**

**Authors’ response**: We agree with the suggestion and have revised the manuscript accordingly.

**See abstract section, page 3, lines 92-94.**

*“Our study revealed that maternal exposure to any form of AN-IPV (physical or sexual) was associated with reduced EIBF. However, no significant association was observed between AN-IPV and EBF at six weeks postpartum, in a large sample of mothers in Ethiopia.”*

**Consistency in Terminology
Ensure consistent use of terms such as “AN-IPV,” “antenatal IPV,” and “IPV during pregnancy” throughout the manuscript.** **AN-IPV**

**Authors’ response**: We have revised the manuscript to ensure consistent use of terminology. The term AN-IPV is now used uniformly throughout the manuscript after its first definition.

**Grammar and Formatting
Minor typographical and formatting inconsistencies appear in tables and headings.
Suggestion: A careful copy-edit is recommended prior to publication.**

**Authors’ response:** The manuscript has been thoroughly copy-edited, and all typographical and formatting inconsistencies have been corrected in the revised version.

**References**

1.	De González AB, Richardson DB, Schubauer-Berigan MK. Statistical Methods in Cancer Research Volume V: Bias Assessment in Case–Control and Cohort Studies for Hazard Identification. 2024.

2.	United Nations Children’s Fund. INTIMATE PARTNER VIOLENCE AND BREASTFEEDING:

A SUMMARY OF THE EVIDENCE BASE [ Access date; June 2, 2025]. Available from: https://www.unicef.org/media/128256/file/UNICEF_GBViE_Breastfeeding_Brief_September_2022.pdf.

3.	Martin‐de‐las‐Heras S, Velasco C, Luna‐del‐Castillo JdD, Khan KS. Breastfeeding avoidance following psychological intimate partner violence during pregnancy: a cohort study and multivariate analysis. BJOG: An International Journal of Obstetrics & Gynaecology. 2019;126(6):778-83.

4.	Khalid N, Zhou Z, Nawaz R. Exclusive breastfeeding and its association with intimate partner violence during pregnancy: analysis from Pakistan demographic and health survey. BMC women's health. 2024;24(1):186.

5.	Olubodun T, Asefa A, Banke-Thomas A, Balogun M, Okafor I, Odukoya O, et al. Maternal exposure to intimate partner violence and breastfeeding practices of children 0–23 months: findings from the 2018 Nigeria demographic and health survey. Journal of Global Health Reports. 2023;7.

6.	Ariyo T, Jiang Q. Intimate partner violence and exclusive breastfeeding of infants: analysis of the 2013 Nigeria demographic and health survey. International breastfeeding journal. 2021;16(1):15.

7.	Kjerulff Madsen F, Holm-Larsen CE, Wu C, Rogathi J, Manongi R, Mushi D, et al. Intimate partner violence and subsequent premature termination of exclusive breastfeeding: A cohort study. PloS one. 2019;14(6):e0217479.

8.	James JP, Taft A, Amir LH, Agius P. Does intimate partner violence impact on women's initiation and duration of breastfeeding? Breastfeeding review. 2014;22(2):11-9.

9.	Wallenborn JT, Cha S, Masho SW. Association between intimate partner violence and breastfeeding duration: results from the 2004-2014 pregnancy risk assessment monitoring system. Journal of Human Lactation. 2018;34(2):233-41.

10.	Pramod DA. Link Between Cortisol Levels and Breastfeeding - Connection and Importance 2024 [Accessed February 19, 2026 ]. Available from: https://www.icliniq.com/articles/womens-health/link-between-cortisol-levels-and-breastfeeding?utm_.

11.	Ziomkiewicz A, Babiszewska M, Apanasewicz A, Piosek M, Wychowaniec P, Cierniak A, et al. Psychosocial stress and cortisol stress reactivity predict breast milk composition. Scientific Reports. 2021;11(1):11576.

12.	Boutet C, Vercueil L, Schelstraete C, Buffin A, Legros J, editors. Oxytocin and maternal stress during the post-partum period. Annales D'endocrinologie; 2006.

13.	Chowdhury AN, Ramakrishna J, Chakraborty AK, Weiss MG. Cultural context and impact of alcohol use in the Sundarban Delta, West Bengal, India. Social Science & Medicine. 2006;63(3):722-31.

14.	Hasselmann MH, Werneck GL, Silva CVCd. Symptoms of postpartum depression and early interruption of exclusive breastfeeding in the first two months of life. Cadernos de saude publica. 2008;24:s341-s52.

15.	Kendall-Tackett K. Breastfeeding and trauma: What lactation specialists need to know. Breastfeeding Medicine. 2020;15(8):495-500.

16.	Tran LM, Nguyen PH, Naved RT, Menon P. Intimate partner violence is associated with poorer maternal mental health and breastfeeding practices in Bangladesh. Health policy and planning. 2020;35(Supplement_1):i19-i29.

17.	Jackson KT, Mantler T, Davidson CA, Siwik E. Intimate Partner Violence and Breastfeeding Outcomes Among Canadian Women: An Exploratory Correlational Study. Maternal and Child Health Journal. 2024;28(10):1737-48.

18.	Cavallé‐Abasolo E, Dikmen‐Yildiz P, Gómez‐Gómez I, Barros‐Martins L, Motrico E. Impact of Traumatic Childbirth and Birth‐Related Posttraumatic Stress Disorder on Breastfeeding Outcomes: A Systematic Review of Longitudinal and Cohort Studies. Birth. 2025;52(4):553-71.

19.	Walters CN, Rakotomanana H, Komakech JJ, Stoecker BJ. Maternal experience of intimate partner violence is associated with suboptimal breastfeeding practices in Malawi, Tanzania, and Zambia: insights from a DHS analysis. International breastfeeding journal. 2021;16(1):20.

20.	Normann AK, Bakiewicz A, Madsen FK, Khan KS, Rasch V, Linde DS. Intimate partner violence and breastfeeding: a systematic review. BMJ open. 2020;10(10):e034153.

21.	Leight J, Wilson N. Intimate partner violence and maternal health services utilization: evidence from 36 National Household Surveys. BMC Public Health. 2021;21(1):405.

22.	Jackson KT, Larose S, Mantler T. Accessing trauma-and violence-informed breastfeeding support from primary care providers among women with histories of intimate partner violence: An exploratory interpretive description study. Canadian Journal of Nursing Research. 2025;57(2):177-87.

23.	Hassen HM. Trends and disparities in ever-breastfeeding practice and early breastfeeding initiation in Ethiopia: a 20-year trend analysis from EDHS datasets. BMC Public Health. 2024;24(1):2558.

24.	Carlin RF, Mathews A, Oden R, Moon RY. The influence of social networks and norms on breastfeeding in African American and Caucasian mothers: A qualitative study. Breastfeeding Medicine. 2019;14(9):640-7.

25.	Aarts C, Kylberg E, Hörnell A, Hofvander Y, Gebre-Medhin M, Greiner T. How exclusive is exclusive breastfeeding? A comparison of data since birth with current status data. International Journal of epidemiology. 2000;29(6):1041-6.

26.	Fenta EH, Yirgu R, Shikur B, Gebreyesus SH. A single 24 h recall overestimates exclusive breastfeeding practices among infants aged less than six months in rural Ethiopia. International breastfeeding journal. 2017;12(1):36.
